# Lost and found: a unique foregut gastrointestinal bullet embolism

**DOI:** 10.1093/jscr/rjae611

**Published:** 2024-09-26

**Authors:** Hunter J Landwehr, Jared B Hinton, Andrew M Loudon, Matthew L Moorman

**Affiliations:** Northeast Ohio Medical University, College of Medicine, 4209 St., OH-44, Rootstown, OH 44272, United States; Northeast Ohio Medical University, College of Medicine, 4209 St., OH-44, Rootstown, OH 44272, United States; Northeast Ohio Medical University, College of Medicine, 4209 St., OH-44, Rootstown, OH 44272, United States; Department of Surgery, University Hospitals, 1110 Euclid Ave., Cleveland, OH 44106, United States; Case Western Reserve University, 9501 Euclid Ave., Cleveland, OH 44106, United States; Northeast Ohio Medical University, College of Medicine, 4209 St., OH-44, Rootstown, OH 44272, United States; Department of Surgery, University Hospitals, 1110 Euclid Ave., Cleveland, OH 44106, United States; Case Western Reserve University, 9501 Euclid Ave., Cleveland, OH 44106, United States

**Keywords:** bullet emboli, gunshot wound oropharynx, penetrating gastrointestinal trauma, trauma surgery

## Abstract

Bullet embolism is a rare phenomenon where a bullet migrates from its original point of entry to a distant site within the body. This brief report describes a case of a bullet embolism entering the gastrointestinal (GI) tract through the posterior oropharynx. The patient initially presented with a gunshot wound to the left scapula, and the bullet was later identified in the GI tract. The patient was managed with a combination of endoscopic techniques and serial imaging, avoiding unnecessary surgical intervention. This case underscores the importance of comprehensive diagnostic strategies and tailored management in GI bullet embolism. It also emphasizes the utility of endoscopy in detecting GI tract injuries and highlights the successful use of non-operative management in specific scenarios.

## Introduction

Bullet embolism is a rare phenomenon where a bullet migrates from its original point of entry to a distant site within the body. Typically, this refers to bullets entering vascular structures, where arterial emboli are twice as common as venous [1]. In a small number of cases, bullets enter and travel within the gastrointestinal (GI) tract. The point of entry has been reported from the oropharynx to the colon [2–6].

GI bullet emboli pose unique clinical challenges. Management of these injuries is not well defined. A projectile in the GI tract can likely be handled similar to a swallowed foreign body (FB). More concerning is the enterotomy resulting from GI tract entry, the location of which is not always apparent on initial imaging. Delayed recognition and treatment of the perforation may lead to serious complications, such as peritonitis or mediastinitis. In some cases, the definitive diagnosis is not made until autopsy [2].

Currently, there are no recommended approaches for detecting or treating these cases. If the site of GI injury is identified below the diaphragm, prompt laparotomy is indicated [7]. Above the diaphragm, the correct approach is less clear, especially in the asymptomatic patient. Minimally invasive techniques for the injured esophagus and non-operative management of oropharyngeal injuries allow for much less morbid therapies.

We present a case highlighting the diagnostic and therapeutic challenges associated with a GI bullet embolism entering the posterior oropharynx. Correct identification of the point of entry as amenable to non-operative treatment avoided unnecessary procedures and morbidity.

## Case report

**Figure 1:**
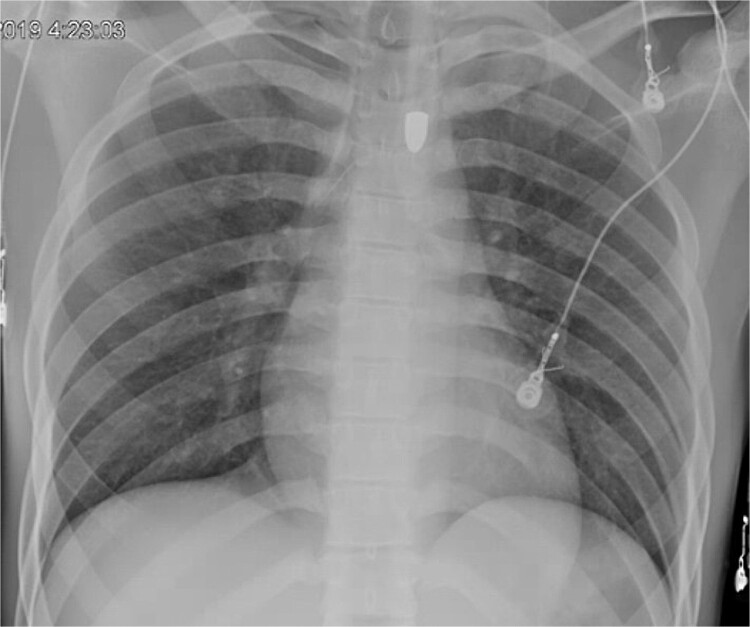
Trauma bay chest X-ray showing retained projectile.

**Figure 2:**
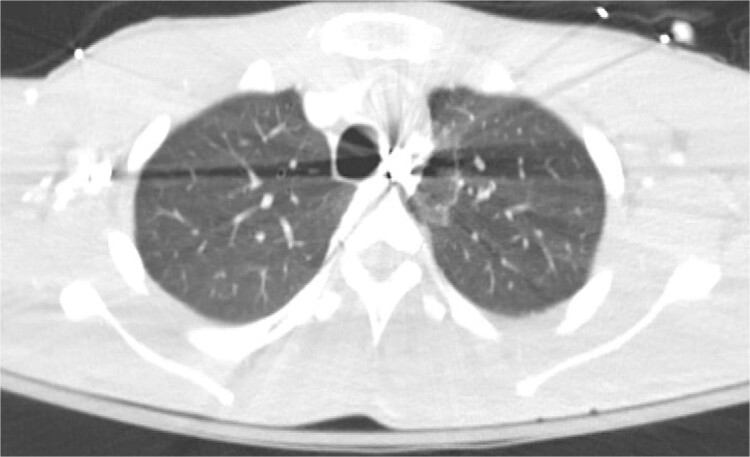
CT angiogram of chest showing retained projectile.

A 21-year-old male with a GSW to the left scapula presented to our Level 1 trauma center. He was stable but had flaccid paralysis and no rectal tone. A chest X-ray showed a bullet in the upper mediastinum (Fig. 1), prompting a computed tomography (CT) angiogram. The CT revealed a C6 cervical spine fracture, epidural hematoma, pneumomediastinum, and the bullet in the mediastinum at approximately the level of T3, but no hemorrhage (Fig. 2). Immediate upper endoscopy and bronchoscopy under general anesthesia were normal. He was extubated and monitored in the trauma ICU.

**Figure 3:**
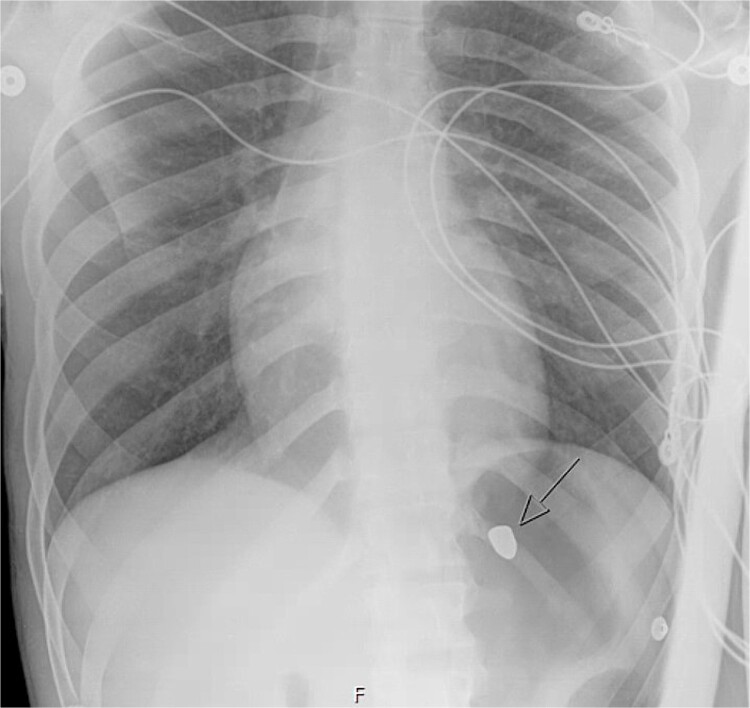
Hospital day 1 chest X-ray.

On hospital day (HD) 1, he developed ARDS and required intubation. He became hypotensive, raising concern for mediastinitis, but repeat imaging showed no new findings except that the bullet had migrated below the diaphragm (Fig. 3). Despite his septic appearance, his condition didn’t improve. He was diagnosed with severe AIDS (CD-4 count <100 cells/mm^3^) and multiple infections (hepatitis A, C, cytomegalovirus, toxoplasmosis, and multi-drug-resistant *Pseudomonas pneumonia*).

**Figure 4:**
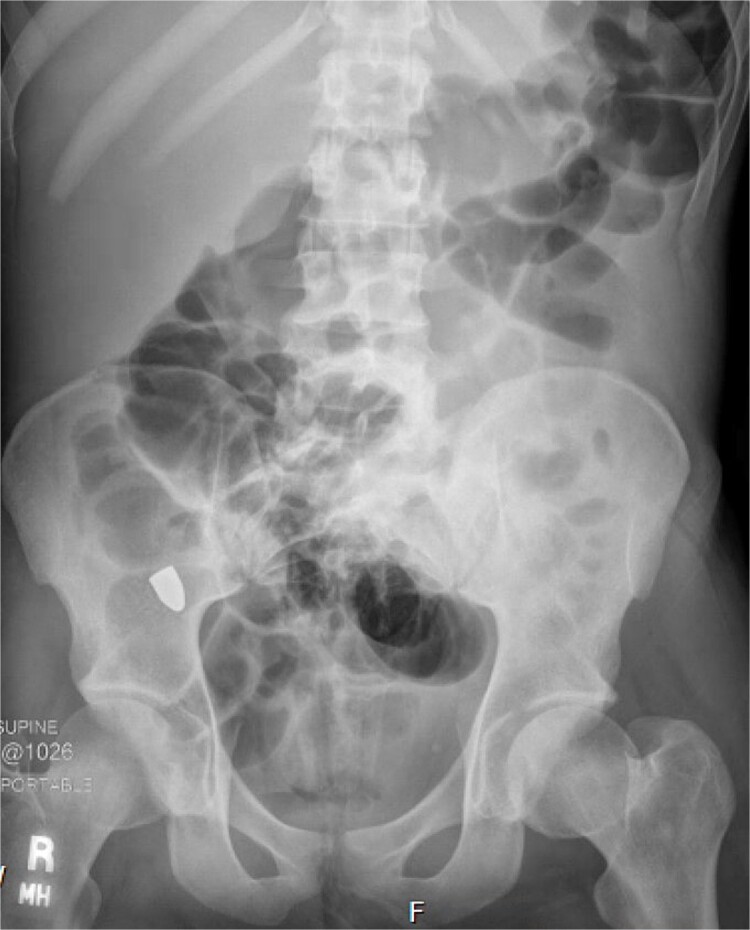
Retained projectile now at the cecum.

A repeat otolaryngology endoscopy exam on HD 2 revealed a 1.5 cm wound in the posterior pharyngeal wall, confirming the suspicion that the bullet entered the posterior of the mouth, was swallowed, and was now migrating down the GI tract. Serial X-rays tracked its transit (Fig. 4). Despite treatment, his condition remained critical. He underwent tracheostomy, gastrostomy, and diaphragmatic pacemaker placement. The patient was discharged to a long-term care facility on HD 22 but succumbed to complications from AIDS and pneumonia months later.

## Discussion

Despite the poor outcome for this patient due to medical comorbidities, a successful strategy was employed for dealing with a bullet embolism. Equally important is the improvement in our understanding of this phenomenon, which we have seen in subsequent patients. In trauma centers with high volumes of firearm injuries, surgeons become skilled at predicting the trajectory of missiles with physical exams. CT scans help confirm these predictions and connect the dots between the GSW and the retained bullet [8]. In cases such as the one presented, the trajectory of the bullet to its initial location is not always clear. Moreover, when bullets move or embolize, the path and appropriate treatment are even more unclear. Injuries in the abdomen are common, as are decisions on intervention. Trauma surgeons are skilled at minimally invasive exploration of the abdomen to ensure no missed injuries and minimize morbidity of the procedure [9]. Injuries above the diaphragm are particularly difficult. Physical exam findings indicating surgery are less clear. While minimally invasive techniques exist for the esophagus, these cases are rare in trauma and less likely to be employed. The higher morbidity of open thoracic surgery makes these treatment decisions much higher stake. Trauma surgeons must be alert for these possibilities and possess the appropriate diagnostic tools to proceed promptly and safely.

Esophagogastroduodenoscopy (EGD) can be helpful in detecting esophageal injuries in penetrating trauma patients, especially when pneumomediastinum is found, as illustrated in our case report. Flowers *et al.* [10] reported a sensitivity of 100%, specificity of 96%, and accuracy of 97% for flexible EGD in detecting such injuries. While laryngoscopy offers rapid assessment, it provides a very limited view of the upper esophagus. Francis *et al.* [11] demonstrated that laryngoscopy missed 33% of injuries. Hence, it should complement rather than replace EGD for suspected high injuries. Esophagrams, with a reported accuracy of 90%, require contrast material to be swallowed, which can be challenging in uncooperative or comatose trauma patients. Therefore, laryngoscopy may serve as an initial evaluation, followed by EGD, then esophagram when feasible. This may help to ensure lesion detection.

Perforation of the esophagus and subsequent soiling of the mediastinum require surgery. Drainage of the mediastinum and possibly the chest is necessary to avoid sepsis. Repair of the esophagus or endoluminal stenting must be done to stop spillage. Asymptomatic injuries to the oropharynx, as in this case, can be managed without intervention. These patients should be monitored for the development of deep space infections. In addition, structural and functional sequelae in the vocal cord and swallowing should be addressed.

A FB in the GI tract will pass naturally in most cases. Serial images can be used to monitor transit through the gut. Stools can be monitored for evacuation [4, 5]. Obstruction or perforation by any FB is possible. Concern may be raised if the object fails to progress, especially in the right lower quadrant at the cecum. Patients developing signs of bowel obstruction or peritonitis warrant exploration [12, 13].
